# Dwarf Mongooses Lose Body Mass in Hot Weather due to Limited Behavioural Plasticity

**DOI:** 10.1002/ece3.71963

**Published:** 2025-09-05

**Authors:** Lauren S. Vane, Josh J. Arbon, Amy Morris‐Drake, Julie M. Kern, Andrew N. Radford

**Affiliations:** ^1^ School of Biological Sciences University of Bristol Bristol UK; ^2^ School of Environmental and Rural Science University of New England Armidale New South Wales Australia

**Keywords:** behavioural responses, body mass gain, climate change, foraging, long‐term data, plasticity

## Abstract

High temperatures associated with climate change can have adverse effects on wildlife, but behavioural plasticity may buffer such negative effects. Using long‐term data from wild dwarf mongooses ( *Helogale parvula* ), we investigated the impact of high temperatures on daily activity patterns, movement and body mass. On hot days (≥ 35°C) compared with matched cooler ones (≤ 33°C), groups emerged from their overnight sleeping burrow and commenced foraging earlier in the morning and arrived at their overnight sleeping burrow later in the evening. However, there was no evidence that the time spent above ground at the burrow, the proportion of time inactive or the distance moved when foraging were altered on hot days. Consequently, the negative effects of high temperatures were not fully mitigated, as both adults and pups gained less body mass on hot days compared with cooler days. This loss was not compensated fully in the day after a hot day, and evening body mass of adults decreased with an increasing number of consecutive hot days. Together, these results suggest that there are potentially escalating consequences of hot weather for wildlife, especially those species that exhibit limited behavioural plasticity, in an ever‐warming world.

## Graphical abstract

Using long‐term data from wild dwarf mongooses (*Helogale parvula*), we demonstrate that on hot days (≥ 35°C) compared to matched cooler ones (≤ 33°C), mongooses emerged and commenced foraging earlier in morning and arrived at and entered their overnight sleeping burrow later in the evening, but we find no further evidence of behavioural plasticity. Consequently, this plasticity was insufficient to offset the costs of hot weather: both adults and pups gained less body mass on hot days, with no evidence of recovery on subsequent cooler days and with increasing losses across consecutive hot days. This highlights the escalating consequences of a warming world for a species with a limited capacity for behavioural plasticity. Image by Shannon Benson.

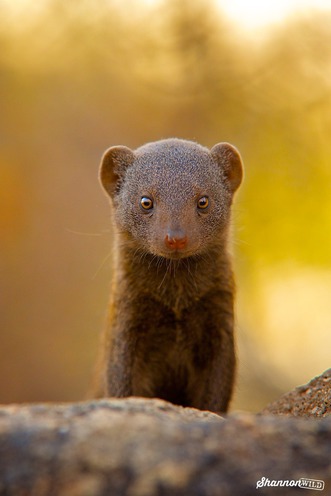

## 1 Introduction

Anthropogenic activities are unequivocally the primary drivers of modern‐day climate change (IPCC 2023) and subsequent declines in biodiversity (Bellard et al. 2012; Franklin et al. 2016). Global surface temperatures have increased faster since 1970 than in any other 50‐year period. High temperatures associated with climate change can have adverse effects across taxa (Parmesan et al. 2000; Pecl et al. 2017), including increased mortality, pathogen outbreak and even species extinction (Pounds et al. 2006; McKechnie and Wolf 2010; Ratnayake et al. 2019; De‐Lima et al. 2022). Hotter conditions are often associated with heat stress and dehydration, which in turn reduce survival (Ratnayake et al. 2019; Thorley et al. 2025). Heat stress also impacts reproduction; for example, gamete function can be compromised if the internal temperature of endotherms exceeds a tolerable limit (Hansen 2009; Jacobs et al. 2024). Small endotherms are particularly vulnerable to high ambient temperatures owing to their high surface area to volume ratio and limited thermal inertia, leading to rapid heat gain when the environmental temperature exceeds that of the body (Withers et al. 2016). However, animals can exhibit behavioural plasticity to lessen the impacts of a changing world (Candolin and Wong 2012).

To mitigate the effects of hot weather, individuals can alter their behaviour in various nonexclusive ways. One strategy is to adjust activity temporally to avoid overlap with the hottest diurnal periods (Levy et al. 2019), as observed in Alpine ibexes ( *Capra ibex* ) (Aublet et al. 2009), whilst some diurnally adapted species, including coruros ( *Spalacopus cyanus* ) and African wild dogs ( *Lycaon pictus* ), are shifting towards nocturnality (Rezende et al. 2003; Rabaiotti and Woodroffe 2019). Another behavioural strategy to combat higher temperatures is to alter habitat use. For instance, an increased use of thermal refugia, such as shaded areas or higher altitudes, is seen in mountain Apollo butterflies (*Parnassius apollo*), moose ( *Alces alces* ) and greater prairie chickens ( *Tympanuchus cupido* ) (Ashton et al. 2009; Alston et al. 2020; Londe et al. 2021). When temperatures are elevated, animals may also spend more time inactive or move less when being active, as found in greater kudus ( *Tragelaphus strepsiceros* ) and American pikas ( *Ochotona princeps* ) (Owen‐Smith 2006; Hall and Chalfoun 2019). However, there may be constraints to plasticity (e.g., a species might not have the sensory capacity to switch to nocturnal activity) or costs to any plasticity exhibited (e.g., being less active may reduce the time available for foraging or result in greater depletion of localised food resources) (Woodroffe et al. 2017; Hall and Chalfoun 2019). Plasticity may also incur energetic costs associated with building and maintaining the required sensory and cognitive machinery (DeWitt et al. 1998; Van Buskirk and Steiner 2009) or elicit maladaptive behavioural responses to novel environmental cues (Robertson et al. 2013; Wong and Candolin 2014). Thus, it is important to investigate not only the behavioural changes seen in response to hotter temperatures but the consequences that could ultimately affect fitness, such as effects on body mass (Van de Ven et al. 2020).

Body mass is an important life‐history trait indicative of both previous resource acquisition and future survival and reproductive prospects (Altmann et al. 1993; Van de Ven et al. 2019). Body mass reductions lessen resilience to resource scarcity (Bright Ross et al. 2021) and hinder thermoregulation during cold weather (Harding et al. 2005), which reduces survival. Body mass also influences reproductive success either directly—for example, by affecting energetic resources available for investment in reproduction (Heldstab et al. 2017)—or indirectly—such as by impacting parental care (Ozgul et al. 2014). Slower offspring development as a consequence of reduced body mass gain negatively affects fitness too (Van de Ven et al. 2019). Importantly, high temperatures can cause reductions in body mass, as documented in southern pied babblers (*Turdoides bicolour*) (du Plessis et al. 2012), white‐plumed honeyeaters (*Ptilotula penicillatus*) and meerkats ( *Suricata suricatta* ) (Paniw et al. 2019). This is likely, at least in part, because the physiological mechanisms underpinning thermoregulation are often associated with greater energetic expenditure, as seen in white‐throated woodrats ( *Neotoma albigula* ) and banded mongooses ( *Mungos mungo* ) (Khera et al. 2023; Ramirez et al. 2022). If homeothermy fails and heat stress ensues, increased protein breakdown into smaller component parts, suppressed appetite due to diversion of resources from growth to thermoregulation and elevated evaporative water loss can all contribute to reduced body mass (Müller and Lojewski 1986; Walsberg 2000; Pearce et al. 2014).

Individuals differ in their responses to high temperature depending on, for instance, size (Genner et al. 2010), body condition (Turko et al. 2020) and age (Grosiak et al. 2020). Younger individuals appear more susceptible than adults to reductions in body mass with increasing temperature (Salaberria et al. 2014; Van de Ven et al. 2020; Khera et al. 2023), often due to reductions in parental care in hot conditions (Cunningham et al. 2013; Wiley and Ridley 2016). Moreover, when temperatures exceed a critical threshold, foraging may be compromised, and thus the ability to regain body mass effectively is limited (du Plessis et al. 2012), and body mass reductions can be exacerbated as the frequency of hot days increases (Cunningham et al. 2013; Gardner et al. 2016). Hence, consideration of age‐related variation, the potential for compensatory recovery, and the cumulative consequences of prolonged heat exposure are fundamental to gain a full understanding of resilience in an ever‐warming world.

Here, we use long‐term data (2013–2022) from a wild population of dwarf mongooses ( *Helogale parvula* ) to investigate how hot daily air temperatures affect behaviour and body mass. Dwarf mongooses are cooperatively breeding, diurnal mammals that live in groups of up to 30 individuals, comprising a dominant breeding pair and nonbreeding subordinate adults of both sexes (Rood 1983; Arbon, Morris‐Drake, Kern, Howell, et al. 2024). Groups use refuges, usually termite mounds, tree trunks or rock piles, in which to sleep at night (Rood 1983). On emergence from their overnight sleeping refuge (hereafter ‘burrow’), group members groom each other in the vicinity (within 5 m) of the burrow (hereafter ‘at the burrow’). They then commence collective foraging excursions around their territory (mean size = 22 ha; Arbon, Morris‐Drake, Kern, Giuggioli, and Radford 2024), which they defend against rival groups (Christensen et al. 2016; Morris‐Drake, Linden, et al. 2021). In the evening, groups generally arrive at a burrow before sunset where they spend time allogrooming before entering the burrow for the night. Wild dwarf mongooses can be habituated to the close presence of human observers, enabling collection of long‐term life‐history, behavioural and body mass data from known individuals and groups in natural conditions.

We began by investigating the impact of high daytime temperature on the activity patterns and movement of dwarf mongoose groups, and the body mass gains of adults and pups, by comparing data from matched pairs of hot and cooler days. We predicted that, to capitalise on cooler parts of the day for foraging, groups would depart earlier from and return later to an overnight burrow on hot days, as well as reduce time spent at the burrow in the morning and evening. We also predicted that, to lessen the risk of heat stress, mongooses would be relatively more inactive and cover less distance once the group left the burrow on hot days compared to cooler ones. If any behavioural plasticity exhibited was sufficient to mitigate negative effects of higher temperatures (e.g., greater thermoregulatory costs), we expected no difference in daily body mass gains between hot and cooler days. However, if any behavioural plasticity was insufficient, then we expected lower body mass gains on hot days compared to cooler ones. Having discovered the latter to be true (see Section 3), we investigated the possibility of compensatory body mass gains following hot days and the cumulative effects of sequences of hot days. We predicted greater body mass gains on cooler days following hot ones compared to cooler days that follow another cooler day, and that the effect of high temperature on body mass would be exacerbated following a greater number of consecutive hot days.

## 2 Methods

### 2.1 Study Site and Population

Data were collected as part of the long‐term Dwarf Mongoose Research Project (DMRP) on Sorabi Rock Lodge Reserve, a private game reserve in Limpopo Province, South Africa (24°11′ S, 30°46′ E). Full details of the study site are in Kern and Radford (2013). Average daily maximum temperature (annual mean ± SE) in this region has risen from 27.7°C ± 0.8°C in 1978 to 29.6°C ± 0.7°C in 2023 (South African Weather Service, unpub. data). Weather conditions during this study were recorded daily on the reserve: rainfall was recorded using a rain gauge; maximum ambient air temperature was recorded using a mercury thermometer suspended in the shade. Study groups (*N* = 12; mean ± SE adult group size = 8.0 ± 0.2; range = 3–17) were habituated to the close presence of human observers (< 5 m proximity on foot) and monitored between 2013 and 2022. Individuals were identifiable via small blonde dye‐marks on their fur and were trained to climb onto an electronic balance scale (accuracy ±1 g) in exchange for a few crumbs of hard‐boiled egg. Mean ± SE body mass of adults (individuals > 1 year) was 245 ± 0.2 g and that of pups (individuals < 1 year) was 187 ± 0.2 g (DMRP unpub. data).

### 2.2 Data Collection

**FIGURE 1:**
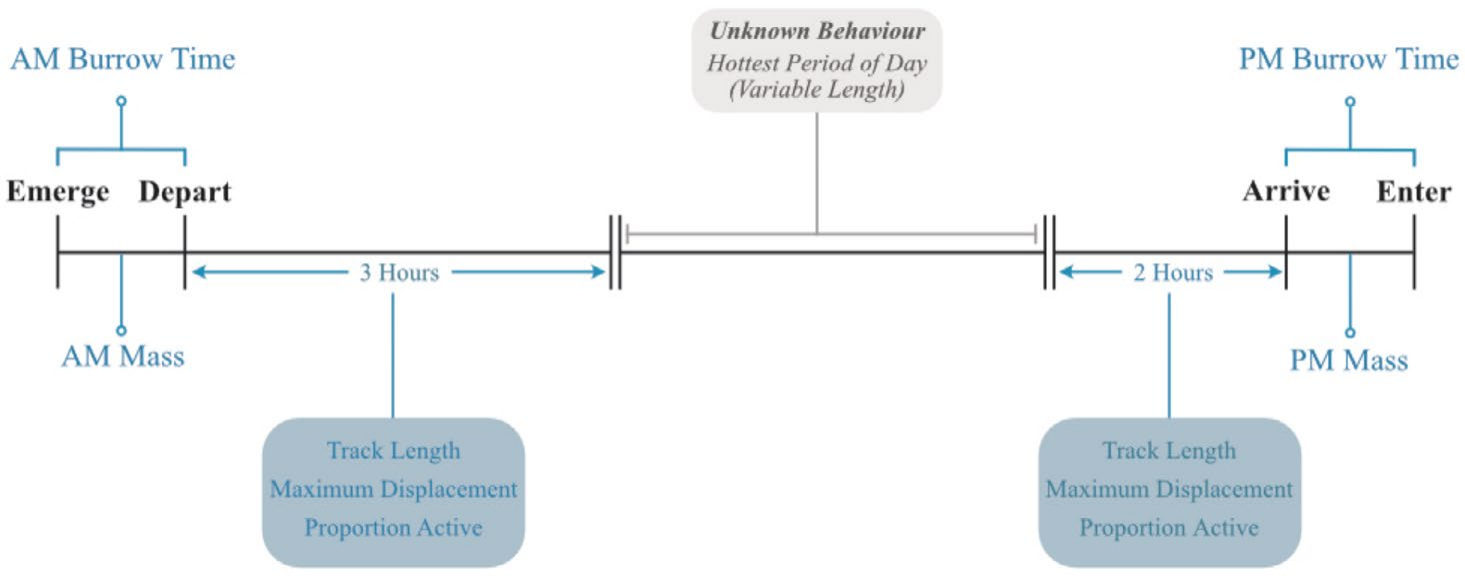
Timeline of data collection relative to dwarf mongoose daily activity. *Emerge*, *Depart*, *Arrive* and *Enter* refer to the timings of behaviour at the sleeping burrow. AM and PM body mass were recorded shortly after emergence and arrival, respectively. GPS data were collected during a 3‐h morning and 2‐h afternoon foraging period (‘activity window’). *Unknown Behaviour* indicates the period during which there was no observer present.

The DMRP comprised a year‐round team of four researchers. New team members were rigorously trained by a Field Manager before collecting data alone, with all data entry carefully checked by both the Field Manager and a UK‐based Data Manager. Each study group was typically observed by a researcher for 2–3 days per week, when behavioural, body mass and life‐history data were collected. Researchers were rotated to ensure equal sampling effort across all groups; all team members visited all groups. Observations were split between a morning and an afternoon session (Figure 1) so that, for health and safety reasons, researchers were not in the field during the hottest part of each day (~11:00–14:00, longer on hotter days). In the summer months, mongoose groups become inactive in the middle of the day, often resting in the shade or retreating below ground; we do not have data on this period. Morning observation sessions commenced when the first individual emerged from their overnight burrow and continued for 3–4 h thereafter. Observers returned to groups ~3 h before the group's predicted return to their night‐time sleeping burrow to commence the afternoon observation session, which concluded when the last individual entered the burrow.

Observers recorded the time by which 50% of group members emerged from the overnight burrow and departed the burrow area in the morning, as well as arrived at and entered the burrow in the evening (Figure 1). When groups were away from a burrow, continuous movement data were collected using a Garmin eTrex10 handheld GPS device (Garmin, Kansas, USA). Observers positioned themselves at the centroid of the group; GPS resolution was generally < 3 m. Observers also took waypoints ca. every 15 min, recording current group activity (foraging, moving or inactive). During every session, full group composition was recorded; pregnancies were recorded during the breeding season. Body masses were obtained from as many group members as possible on emergence from the overnight burrow in the morning and on arrival at a burrow in the evening.

### 2.3 Behaviour Data Extraction and Statistical Analysis

To investigate the impact of a high temperature day on group activity and movement, we conducted paired data extraction and analysis. The matched analyses controlled for potential confounding variables such as group identity and size, food availability and specific year. We initially identified days characterised by a maximum ambient temperature ≥ 35°C, hereafter ‘hot days’. We then identified potential paired ‘cooler days’ by collating all days ≤ 33°C within a 5‐day window either side of a hot day. To be included in the analyses, observations on paired days needed to have been conducted on the same study group with identical group composition. There also had to have been no rain (as mongooses retreat underground in heavy downpours) or intergroup interactions (as these are major disruptions to other behaviours). For hot days with multiple potential cooler counterparts, we chose the combination that, in order, maximised the number of total pairs, maximised the difference in temperature within pairs, minimised the variation in temperature difference between pairs, minimised the time difference within pairs, and finally minimised the variation in time difference between pairs. If all those parameters were equal, we chose combinations at random. In our final paired dataset, hot days had a mean ± SE temperature of 36.5°C ± 0.1°C, while cooler days were 28.9°C ± 0.2°C (Table 1).

**TABLE 1:** Replication statement.

Response metric	Scale of inference	Scale at which the factor of interest is applied	Number of replicates at the appropriate scale
Activity timings	Social group	Local population	*N*_Groups_ = 12, *N*_Pairs_ = 96–130
Movement	Social group	Local population	*N*_Groups_ = 10–11, *N*_Pairs_ = 28–74
Body mass	Social group	Local population	*N*_Groups_ = 7–12, *N*_Pairs_ = 21–41, *N*_Days_ = 115–274

*Note:* *N*_Groups_ represents the number of social groups sampled, *N*_Pairs_ is the number of paired hot and cooler days analysed, and *N*_Days_ is the number of days analysed within unpaired analyses.

We conducted all statistical analyses using R version 4.4.0 (R Core Team 2022). We fit linear mixed models (LMMs) and generalised linear mixed models (GLMMs) with the packages ‘lme4’ (Bates et al. 2015) and ‘glmmTMB’ (Brooks et al. 2017). We checked all datasets for outliers, removing any data points with a *z* score of > 3. Model diagnostics were used to inform model error structure and link function using the ‘DHARMa’ package (Hartig 2017), to ensure that the associated assumptions (normality of residuals and homogeneity of variance) were satisfied. If singular model fit was obtained (e.g., due to too few data points from particular groups to allow sufficient estimate of every level of the random effect), the ‘blmer/bglmer’ Bayesian wrapper functions from the ‘blme’ package were used (Chung et al. 2013). In such cases, the default Wishart covariance prior provides a weakly informative prior to aid model fitting and ensure the preservation of the a priori selected random effects structure; this is reflected in model table legends. We calculated all *p* values via likelihood ratio tests (LRTs) comparing the full model with a model from which the fixed factor of interest was removed (Crawley 2005). If a model contained an interaction effect, it was removed if nonsignificant to aid interpretation of main effects.

For all models examining behavioural response metrics, we fitted temperature class (hot or cooler day) as a fixed effect. To account for paired data points and multiple pairs of data from the same group, pair identity and group identity were fitted as random effects, with pair nested within group. For each cluster of analyses (e.g., the three response measures at the burrow in the morning), we applied a sequential correction for multiple testing; that is, we took the smallest *p* value in the cluster and used an alpha value of 0.05 divided by the total number of response metrics, repeating this process for each remaining result and dividing by the number of remaining response metrics under consideration. All presented significant results are significant following this correction procedure.

#### 2.3.1 Group Behaviour at the Burrow

To maximise our sample size, we extracted morning and evening burrow activity data independently. To determine the effect of high temperature on behaviour at the burrow in the morning, we conducted separate LMMs for the two response variables: time by which 50% of group members had emerged from the burrow and time by which 50% of group members had departed to commence foraging. We calculated times relative to sunrise to account for seasonal variation in daylength. We also used a GLMM (Poisson error structure and log link function) to examine the time spent at the burrow in the morning, defined as the time between emergence and departure.

To determine the effect of high temperature on behaviour at the burrow in the evening, we conducted a LMM on the time by which 50% of group members had arrived at the burrow and a LMM on the log‐transformed time by which 50% of group members had entered the burrow. Times were calculated relative to sunset. Due to instances when groups arrived at or entered the burrow after sunset, a constant was applied to all times to eliminate negative values and thus enable fitting of a negative binomial model. We used a LMM to examine the square‐root‐transformed time spent at the burrow area in the evening, defined as the time between arrival and entering.

#### 2.3.2 Group Movement

To determine how high temperature affects group movement, we analysed three metrics during a 3‐h period post‐departure from the burrow area in the morning and a 2‐h period preceding arrival at the burrow in the evening (hereafter ‘activity windows’). Due to the peak of daily temperatures being closer to sunset than sunrise, DMRP protocol resulted in a longer morning than afternoon window to ensure the safety of researchers in high temperatures. All metrics were analysed for morning and afternoon activity windows separately, as paired data were only available for both windows on a subset of days.

The effect of high temperature on the proportion of time spent inactive during each activity window (as determined from the waypoint data) was analysed using separate GLMMs (binomial structure and log link function); a correction for zero inflation was added to models on data from the PM activity window. We also determined the effect of high temperature on the total distance travelled and on maximum displacement distance during each activity window (all with LMMs). All GPS data were subsampled to a resolution of 1 fix per minute to standardise sampling rate across devices and years; 1 min represents a balance between minimising observer and GPS error‐based movements while maximising resolution of group movement. From that, total distance was calculated as the sum of distances between all fixes, while maximum displacement distance was the maximum linear distance between the point of origin and all other fixes.

### 2.4 Body Mass Data Extraction and Statistical Analysis

To investigate the impact of a high temperature day on body mass gains, we conducted paired data extraction and analysis as for the behavioural data (above). For individuals weighed in both the morning and evening sessions on the same day, we calculated the mean proportional change (relative to morning mass) across all available adults and pups within a group each day. Only pairs of hot and cooler days for which there was at least one individual weighed on both were considered for analysis. We ran separate LMMs on the mean daily proportional body mass change for adults and pups because there were occurrences where we had adult data but no pup data, and we wished to maximise the sample size for both. While observers always ensured the maximum number of individuals weighed per observation session, we used proportional, as opposed to absolute, body mass change to account for variation in the identity of individuals weighed between paired days, as well as differing baseline weights of group members.

After identifying that dwarf mongoose body mass gain is negatively affected on hot compared with cooler days (see Results), we conducted two further body mass analyses. First, we investigated if there is a compensatory increase in body mass gain after a hot day. From the full database, we identified cooler days which either followed a hot day (‘post‐hot’) or control cooler days which followed another cooler day (‘post‐cooler’). We assessed the proportional change in body mass between the morning and evening for both adults and pups on post‐hot versus post‐cooler days. Second, we examined the cumulative effects of consecutive hot days. From the full database, we identified occurrences of a hot day preceded by two cooler days (‘one’) and three consecutive hot days (‘three’). We intended to include occurrences of two consecutive hot days, but the sample size of this category was half that for one and three consecutive hot days, and thus was omitted. We analysed absolute evening body mass for adults on the final day of each sequence to investigate any cumulative effects; there were insufficient pup data for this analysis. For both the compensatory and cumulative analyses, the increased specificity in data filtering prevented us from using a matched approach. To minimise confounding effects on body mass, we excluded days with rain or intergroup interactions, and those on which there was a change in group composition during the day. We focused on days from the breeding season (when the vast majority of hot days occur) but excluded data from pregnant females. Only days for which there were at least two individuals weighed were considered for analysis.

For the models examining a potential compensatory increase in body mass gain, we included day type (post‐hot or post‐cooler) as a fixed effect. For the model examining cumulative effects, we included the number of consecutive hot days (one or three) as a fixed effect. In all models, we also included group size and its interaction with day type or number of consecutive hot days as fixed effects. Normalised difference vegetation index (NDVI) was included as a fixed effect to account for variation in landscape greenness and therefore productivity (Pettorelli et al. 2005; Arbon, Morris‐Drake, Kern, Giuggioli, and Radford 2024; Arbon, Morris‐Drake, Kern, Howell, et al. 2024). We obtained NDVI data from the MODIS product ‘MOD13Q1’ (Didan 2021) taken at 16‐day, 250 m by 250 m resolution, using functions from the ‘MODISTools’ R package (Hukens 2023). Mean NDVI per day for the study site was obtained from the closest available date and then scaled. Group identity and specific season (e.g., 2022 breeding season) were set as random effects in all models.

## 3 Results

### 3.1 Group Behaviour at the Burrow

**FIGURE 2:**
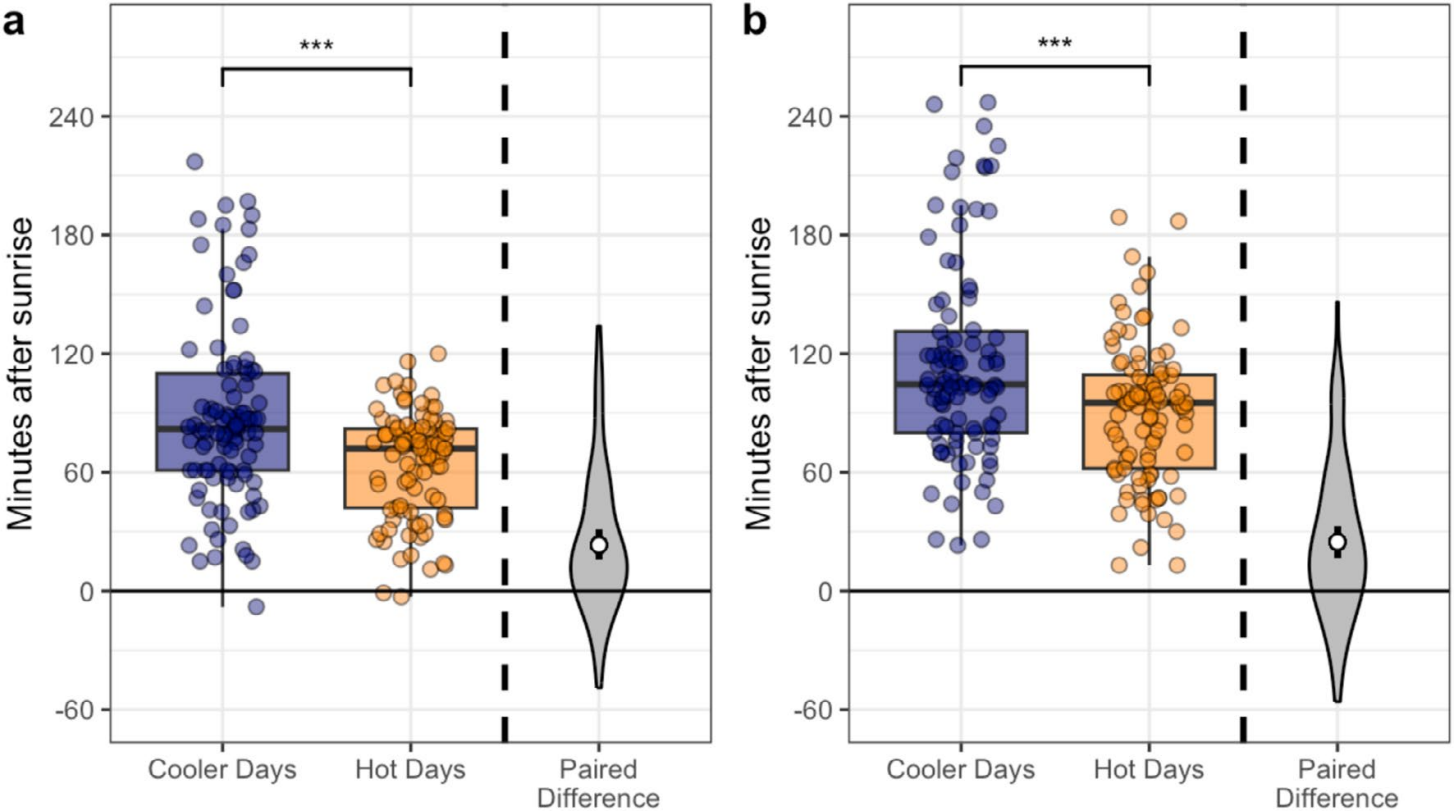
The impact of temperature (cooler—blue; hot—orange) on the (a) emergence time from the overnight burrow relative to sunrise (*N*_Pairs_ = 97), and (b) departure time from the overnight burrow area relative to sunrise (*N*_Pairs_ = 96). Boxplots denote median and interquartile range (IQR), whiskers denote 1.5× IQR. Data points are jittered for ease of viewing. ‘Paired difference’ shows difference between matched pairs of days; white points and arms denote mean and 95% CI of paired difference. Positive difference values indicate behaviour occurred earlier on a hot day than a cooler day, and negative values indicate behaviour occurred later on a hot day than a cooler day. ***p < 0.001.

In the morning, emergence from the overnight burrow was 23.4 ± 37.1 min (mean ± SD) earlier on hot days than cooler days (LRT: *p* < 0.001; Table 2a; Figure 2a). Similarly, groups departed the burrow area to go foraging 24.8 ± 39.0 min earlier on hot days than cooler days (*p* < 0.001; Table 2b; Figure 2b). However, the time spent at the burrow in the morning did not differ significantly between hot (25.2 ± 18.1 min) and cooler (26.7 ± 19.4 min) days, although there was a trend for reduced time on mornings of hot days (*p* = 0.052; Table 2c).

**TABLE 2:** Output from mixed‐effects models (LMMs unless otherwise stated) investigating the impact of high temperature on (a) emergence time from the overnight burrow relative to sunrise, (b) departure time from the burrow relative to sunrise, (c) time spent at the burrow in the morning (GLMM with Poisson error structure and log link function), (d) arrival time at the burrow area relative to sunset, (e) entrance time into the overnight burrow relative to sunset, and (f) time spent at the burrow in the evening.

Fixed effect	Effect	95% CI	*χ* ^2^	df	*p*
(a) Emergence time relative to sunrise (*N*_Pairs_ = 97)					
Intercept	87.67	80.33–95.31			
TemperatureClass	−23.39	−30.71 to −15.87	32.47	1	< 0.001
*Pair:Group*	*27.58*	*21.05–33.02*			
*Group*	*7.04*	*0.00–10.26*			
(b) Departure time relative to sunrise (*N*_Pairs_ = 96)					
Intercept	114.69	106.15–123.78			
TemperatureClass	−24.77	−32.62 to −16.93	32.78	1	< 0.001
*Pair:Group*	*34.71*	27.84–41.22			
*Group*	*9.57*	*0.00–13.75*			
(c) Time at the morning burrow (*N*_Pairs_ = 96)					
Intercept	3.15	2.97–3.31			
TemperatureClass	−0.06	−0.11–0.00	3.77	1	0.052
*Pair:Group*	*0.48*	*0.40–0.57*			
*Group*	*0.16*	*0.00–0.41*			
(d) Arrival time relative to sunset (*N*_Pairs_ = 127)					
Intercept	39.97	35.63–44.14			
TemperatureClass	−12.55	−17.74 to −7.36	20.94	1	< 0.001
*Pair:Group*	*11.54*	*5.87–15.66*			
*Group*	*0.11*	*0.00–6.33*			
(e) Entrance time relative to sunset (*N*_Pairs_ = 129)					
Intercept	3.65	3.56–3.74			
TemperatureClass	−0.31	−0.44 to −0.18	21.09	1	< 0.001
*Pair:Group*	*0.16*	*0.00–0.24*			
*Group*	*0.07*	*0.00–0.11*			
(f) Time at the evening burrow (*N*_Pairs_ = 130)					
Intercept	4.06	3.75–4.36			
TemperatureClass	−0.01	*−0.44–0.43*	*8e* ^*−4*^	1	0.977
*Pair:Group*	*0.49*	*0.00–0.75*			
*Group*	*0.21*	*0.00–0.31*			

*Note:* Bayesian wrapper applied to models a, b, e and f to aid estimation of random effect parameters. Models included temperature class (hot, cooler) as a fixed effect, with cooler set as the reference level, and paired days (pair) nested within group identity as random effects. Variance (SD) for the random effects is reported in italics. Sample sizes refer to the number of pairs of hot and cooler days analysed. For all models *N*_Groups_ = 12.

**FIGURE 3:**
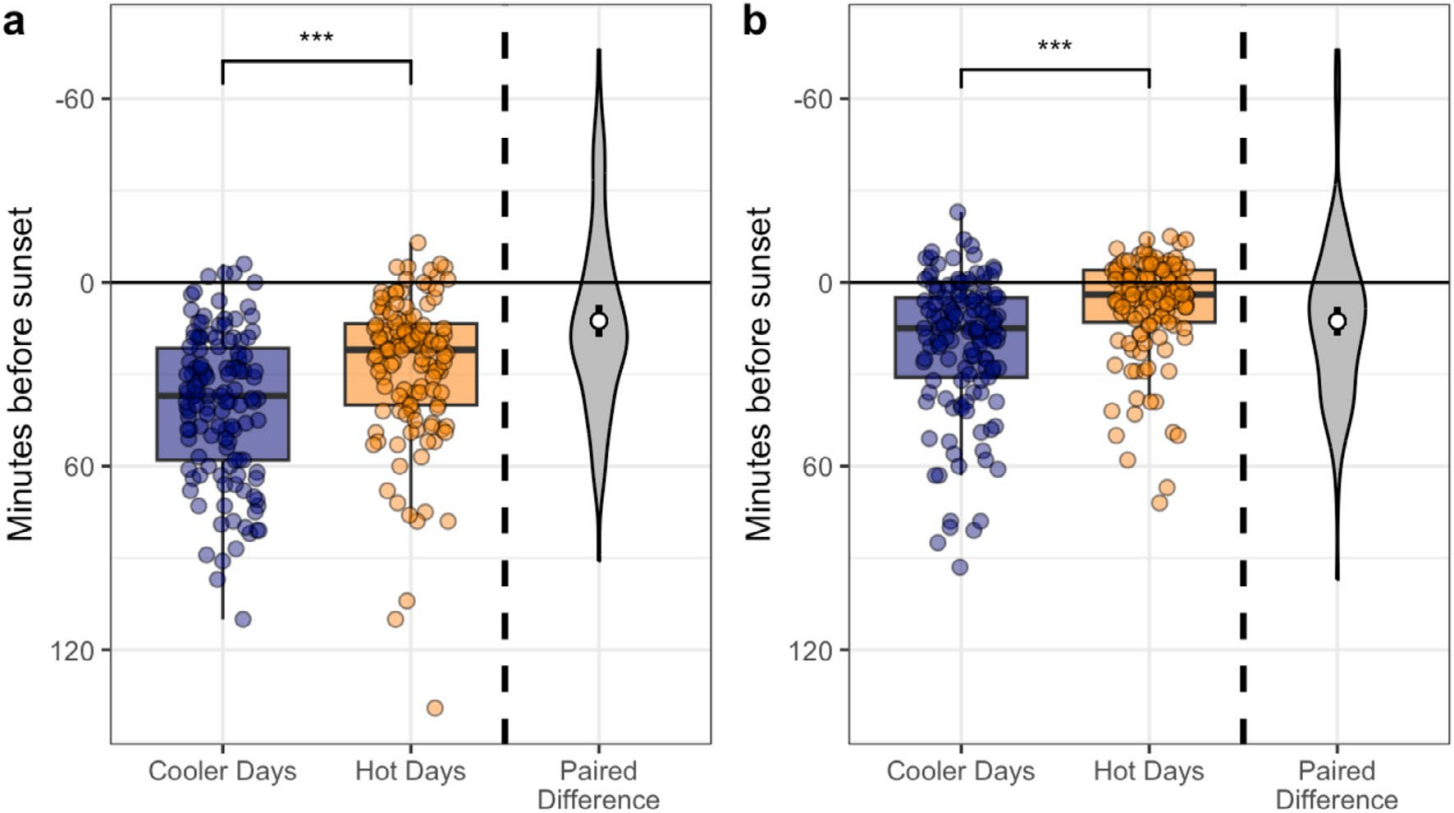
Impact of temperature (cooler—blue; hot—orange) on the (a) arrival time at the night‐time burrow relative to sunset (*N*_Pairs_ = 127), and (b) entrance time into the night‐time burrow relative to sunset (*N*_Pairs_ = 129). Boxplots denote median and inter‐quartile range (IQR), whiskers denote 1.5× IQR. Data points are jittered for ease of viewing. ‘Paired difference’ shows difference between matched pairs of days; white points and arms denote mean and 95% CI of paired difference. Positive difference values indicate behaviour occurred earlier on a hot day than a cooler day, and negative values indicate behaviour occurred later on a hot day than a cooler day. ***p < 0.001.

In the evening, groups arrived at the burrow area 12.6 ± 29.7 min (mean ± SD) later on hot days than cooler days (LRT: *p* < 0.001; Table 2d; Figure 3a). Groups entered the burrow 12.7 ± 26.4 min later on hot days than cooler days (*p* < 0.001; Table 2e; Figure 3b). The time spent at the burrow in the evening did not differ significantly between hot (19.9 ± 15.4 min) and cooler (19.3 ± 15.1 min) days (*p* = 0.977; Table 2f).

### 3.2 Group Movement

The proportion of each activity window that groups spent inactive did not differ significantly between hot and cooler days in the morning (mean ± SD, hot: 27% ± 26%, cooler: 27% ± 31%; LRT: *p* = 0.25; Table 3a), or the afternoon (hot: 44% ± 44%, cooler: 34% ± 42%; *p* = 0.34; Table 3b). Groups did not travel a significantly different total distance on hot versus cooler days in the morning (hot: 1033 ± 403 m, cooler: 990 ± 378 m; *p* = 0.58; Table 3c) or afternoon (hot: 544 ± 219 m, cooler: 611 ± 346 m; *p* = 0.53; Table 3d). There was also no significant difference between hot and cooler days in the maximum displacement distance in the morning (hot: 239 ± 126 m, cooler: 300 ± 174 m; *p* = 0.24; Table 3e) or afternoon (hot: 172 ± 110 m, cooler: 149 ± 68 m; *p* = 0.21; Table 3f).

**TABLE 3:** Output from mixed‐effects models (LMMs unless otherwise stated) investigating the impact of high temperature on: The proportion of time spent inactive in the (a) morning and (b) afternoon (both GLMMs with binomial structure and log link function); total distance travelled in the (c) morning and (d) afternoon; and maximum displacement distance in the (e) morning and (f) afternoon.

Fixed effect	Effect	95% CI	*χ* ^2^	df	*p*
(a) Proportion of time spent inactive in the morning (*N*_Pairs_ = 70, *N*_Groups_ = 11)					
Intercept	−1.22	−1.52 to −0.88			
TemperatureClass	0.15	−0.10–0.40	1.30	1	0.25
*Pair:Group*	*0.74*	*0.55–0.99*			
*Group*	*0.21*	*0.00–0.59*			
(b) Proportion of time spent inactive in the afternoon (*N*_Pairs_ = 75, *N*_Groups_ = 11)					
Intercept	−0.86	−1.34 to −0.38			
TemperatureClass	0.20	−0.21–0.62	0.91	1	0.34
*Pair:Group*	*0.66*	*0.43–1.01*			
*Group*	*0.41*	*0.13–1.30*			
(c) Total distance travelled in the morning (*N*_Pairs_ = 29, *N*_Groups_ = 10)					
Intercept	1011.22	836.78–1197.93			
TemperatureClass	43.18	−115.24–201.60	0.30	1	0.58
*Pair:Group*	*186.80*	*0.00–372.78*			
*Group*	*178.10*	*0.00–354.26*			
(d) Total distance travelled in the afternoon (*N*_Pairs_ = 37, *N*_Groups_ = 10)					
Intercept	565.39	478.87–650.43			
TemperatureClass	−20.78	−87.35–45.79	0.39	1	0.53
*Pair:Group*	*158.33*	*102.64–226.25*			
*Group*	*66.27*	*0.00–166.37*			
(e) Maximum displacement distance in the morning (*N*_Pairs_ = 28, *N*_Groups_ = 10)					
Intercept	277.03	230.63–322.86			
TemperatureClass	−36.58	−98.88–25.72	1.38	1	0.24
*Pair:Group*	*61.59*	*0.00–93.68*			
*Group*	*46.44*	*0.00–69.46*			
(f) Maximum displacement distance in the afternoon (*N*_Pairs_ = 38, *N*_Groups_ = 10)					
Intercept	148.58	116.85–178.98			
TemperatureClass	22.30	−13.31–57.92	1.55	1	0.21
*Pair:Group*	*45.80*	*0.00–75.12*			
*Group*	*14.30*	*0.00–48.96*			

*Note:* Correction for zero inflation applied to model b, Bayesian wrapper applied to model e to aid estimation of random effect parameters. Models included temperature class (hot, cooler) as a fixed effect, with cooler set as the reference level, and paired days (pair) nested within group identity as random effects. Variance (SD) for the random effects is reported in italics. Sample sizes refer to the number of pairs of hot and cooler days analysed, as well as the number of groups from which those pairs of data arose.

### 3.3 Body Mass

**FIGURE 4:**
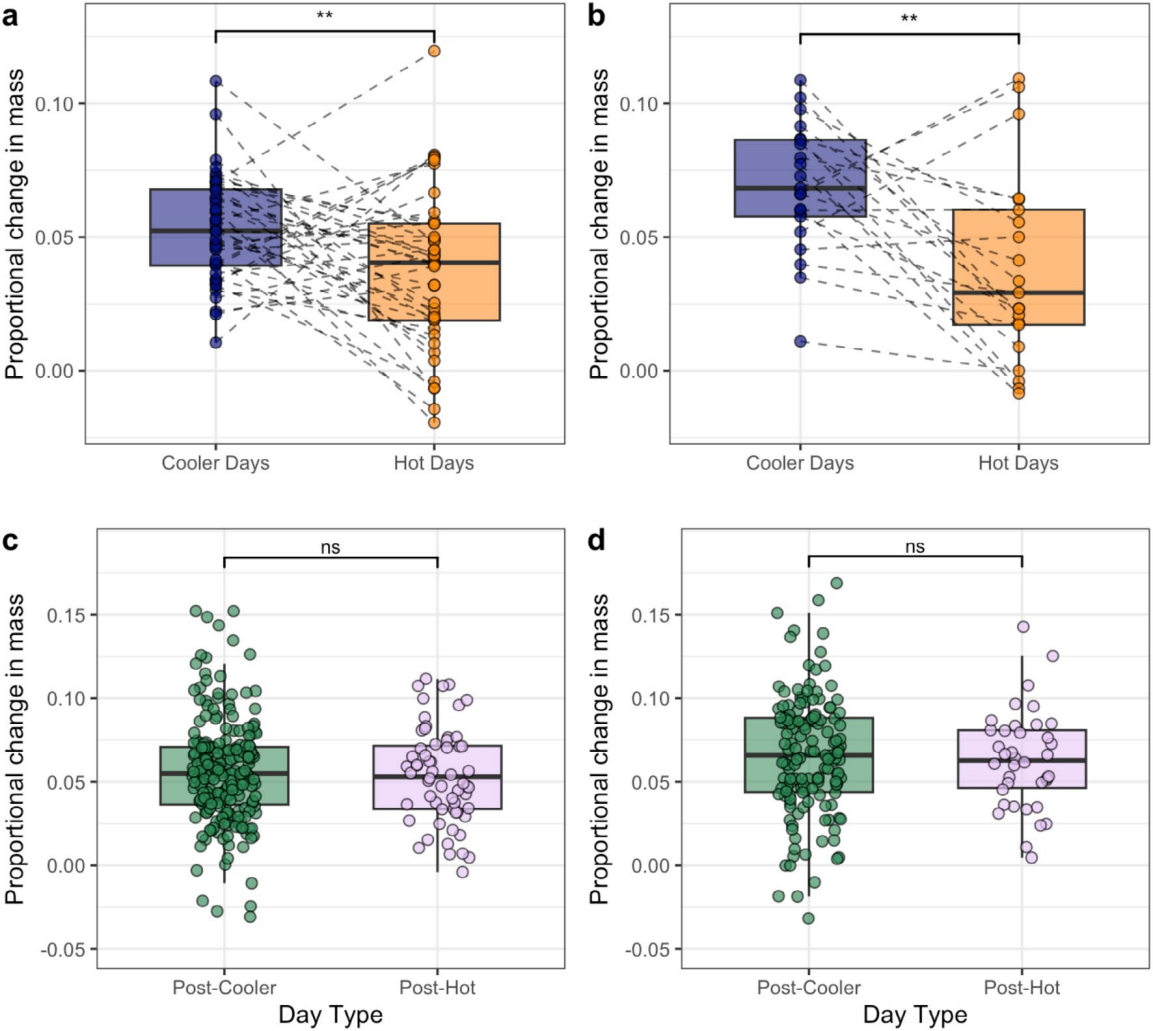
Impact of temperature (cooler—blue; hot—orange) on the change in proportional body mass between morning and evening for (a) adults (*N*_Pairs_ = 41) and (b) pups (*N*_Pairs_ = 21). Proportional body mass gain on a cooler day after a hot day (post‐hot—purple) compared with the day after another cooler day (post‐cooler—green) in (c) adults (*N*_Days_ = 274) and (d) pups (*N*_Days_ = 181). Boxplots denote median and interquartile range (IQR), whiskers denote 1.5× IQR. Dashed lines in (a) and (b) connect paired observation days; data points with the same value may overlap. Data points in (c) and (d) jittered for ease of viewing. **p < 0.01, ^ns^ *p* > 0.05.

Dwarf mongoose adults gained 1.6% ± 2.9% (mean ± SD) less body mass between the morning and evening on hot days compared with cooler days (LRT: *p* = 0.006; Table 4a; Figure 4a). Similarly, dwarf mongoose pups gained 3.1% ± 3.6% less body mass between the morning and evening on hot days compared with cooler days (*p* = 0.002; Table 4b; Figure 4b). There was no evidence for a full compensatory increase in body mass gain the day after a hot day in either adults (*p* = 0.49; Table 5a; Figure 4c) or pups (*p* = 0.53; Table 5b; Figure 4d). To confirm that this absence of significant effects was not due to the smaller unpaired sample, we conducted a permutation‐based analysis to estimate the minimum detection threshold of the linear mixed‐effects models that examined the potential compensatory increases in body mass. For each permutation (*N* = 1000), a subset of rows (number equal to the mean sample size across all levels of the response variable) was randomly selected and a constant was applied to the response variable. We then modelled both the original subset and the shifted subset. Models fitted were otherwise identical in structure to those within the main analyses, and *p* values were obtained using LRTs as explained in Section 2. The minimum detection threshold was then defined as the smallest systematic shift from which a significant difference (*p* < 0.05) between the subsample and the shifted subsample could be reliably detected (> 95% of all permutations). We can confidently detect body mass differences of 0.97% for adults and 1.10% for pups. Hence, our models would provide sufficient resolution to detect compensations of approximately 2/3 and 1/3 of the reduction in daily body mass gain found within the paired analysis.

**TABLE 4:** Output from linear mixed‐effects models investigating the impact of high temperature on the daily body mass gain of dwarf mongoose (a) adults and (b) pups.

Fixed effect	Effect	95% CI	*χ* ^2^	df	*p*
(a) Adult proportional body mass change (*N*_Pairs_ = 41, *N*_Groups_ = 10)					
Intercept	0.05	0.04–0.06			
TemperatureClass	−0.02	−0.03 to −0.01	7.71	1	0.006
*Pair:Group*	*0.01*	*0.00–0.01*			
*Group*	*0.01*	*0.00–0.01*			
(b) Pup proportional body mass change (*N*_Pairs_ = 21, *N*_Groups_ = 7)					
Intercept	0.07	0.06–0.08			
TemperatureClass	−0.03	−0.05 to −0.01	10.04	1	0.002
*Pair:Group*	*0.02*	*0.00–0.02*			
*Group*	*0.01*	*0.00–0.01*			

*Note:* Bayesian wrapper applied to both models to aid estimation of random effect parameters. Models included temperature class (hot, cooler) as a fixed effect, with cooler set as the reference level, and paired days (pair) nested within group identity as random effects. Variance (SD) for the random effects is reported in italics. Sample sizes refer to the number of pairs of hot and cooler days analysed, as well as the number of groups from which those pairs of data arose.

**TABLE 5:** Output from linear mixed‐effects models examining potential compensatory body mass gain on a cooler day after a hot day (post‐hot) compared with control cooler days after another cooler day (post‐cooler). Models analyse proportional body mass change between morning and evening in dwarf mongoose (a) adults and (b) pups.

Factor	Effect	95% CI	*χ* ^2^	df	*p*
(a) Adult proportional body mass change (*N*_Days_ = 274, *N*_Groups_ = 12)					
Intercept	0.05	0.04–0.06			
Day type	0.00	−0.01–0.01	0.47	1	0.49
Group size	0.00	0.00–0.00	0.46	1	0.50
Group size: Day type			0.59	1	0.44
NDVI	0.01	0.00–0.01	1.86	1	0.40
*Season*	*0.01*	*0.00–0.01*			
*Group*	*0.00*	*0.00–0.01*			
(b) Pup proportional body mass change (*N*_Days_ = 181, *N*_Groups_ = 9)					
Intercept	0.07	0.05–0.09			
Day type	0.00	−0.02–0.01	0.40	1	0.53
Group size	0.00	0.00–0.00	0.95	1	0.33
Group size: Day type			0.18	1	0.67
NDVI	0.01	0.00–0.01	3.23	1	0.07
*Season*	*0.01*	0.00–0.02			
*Group*	*0.00*	0.00–0.01			

*Note:* Models included group size, NDVI and day type (post‐hot, post‐cooler) as fixed effects, with post‐cooler set as the reference level, and season and group as random effects. Variance (SD) for the random effects is reported in italics. Bayesian wrapper applied to both models to aid estimation of random effect parameters. Sample sizes refer to the number of days analysed, as well as the number of groups from which those data arose.

**FIGURE 5:**
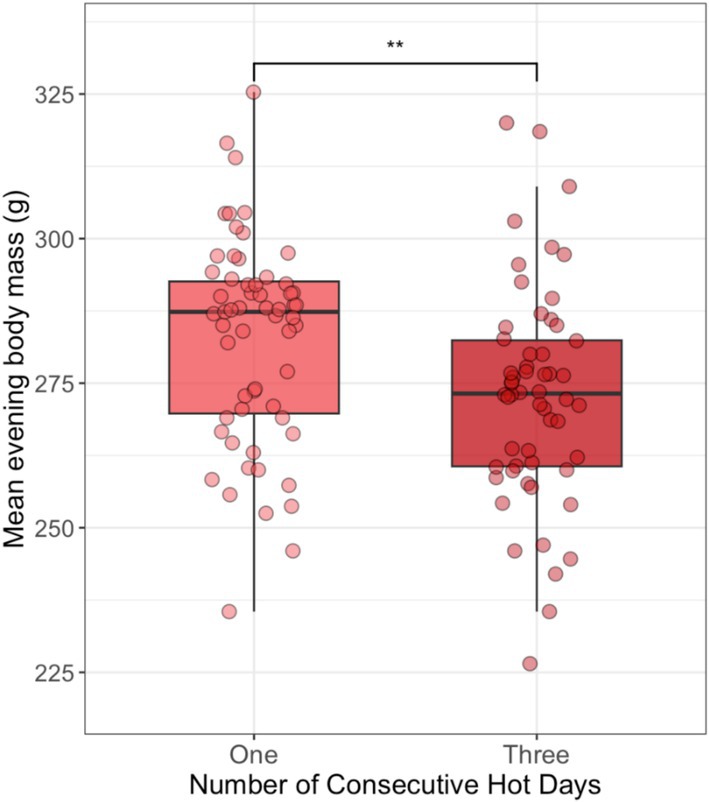
Impact of different numbers of consecutive hot days (one or three) on the evening body mass of dwarf mongoose adults (*N*_Days_ = 115). Boxplots denote median and interquartile range (IQR), whiskers denote 1.5× IQR. Data points jittered for ease of viewing. **p < 0.01.

After controlling for a positive effect of habitat greenness (NDVI; Table 6), dwarf mongoose adults were 3.8% lighter (mean difference ± SE: 10.3 ± 3.3 g) after three consecutive hot days when compared to one hot day (LRT: *p* = 0.002; Figure 5).

**TABLE 6:** Output from linear mixed‐effects model investigating the impact of the number of consecutive hot days (*N* Hot Days) on mean evening body mass of dwarf mongoose adults.

Factor	Effect	95% CI	*χ* ^2^	df	*p*
Mean evening body mass in adults (*N*_Days_ = 115, *N*_Groups_ = 12)					
Intercept	275.26	262.85–287.76			
*N* Hot days: Three	−10.32	−17.24 to −3.83	9.35	1	0.002
Group size	0.15	−0.84–1.12	0.05	1	0.816
Group size: *N* hot days			0.17	1	0.677
NDVI	20.13	8.71–31.09	11.71	1	< 0.001
*Season*	*6.17*	*0.00–13.01*			
*Group*	*3.08*	*0.00–9.33*			

*Note:* Model included group size, NDVI and the number of consecutive hot days (one or three) as fixed effects, with one set as the reference level, and season and group included as random effects. Variance (SD) for the random effects is reported in italics. Sample size refers to the number of days analysed, as well as the number of groups from which those data arose.

## 4 Discussion

Using long‐term data from a wild population, we found little evidence that dwarf mongooses exhibit behavioural plasticity in hot weather; consequently, there were negative effects on body mass. On hot days (≥ 35°C) compared with matched cooler ones (≤ 33°C), groups emerged from overnight burrows and commenced daytime foraging earlier in the morning and arrived at and entered burrows later in the evening. But, contrary to predictions, time spent above ground at the burrow in both the morning and evening, as well as time spent inactive, distance travelled and maximum displacement distance during foraging excursions did not differ significantly between hot and cooler days. Likely related to the limited behavioural changes, both adults and pups gained less body mass on hot days compared with cooler days. There was also no evidence of compensatory increases in body mass gain the day after a hot day, while the evening body mass of adults decreased with an increasing number of consecutive hot days. Together, these results suggest that dwarf mongooses may not be able to change their behaviour sufficiently to mitigate the potential fitness consequences of hot weather. Moreover, a lack of immediate compensation and a cumulative effect of hot‐day sequences suggests that there could be increasing problems for wildlife in an ever‐warming world.

The reduced daily body‐mass gains of both adult and pup dwarf mongooses on hot days are consistent with previous studies on meerkats and pied babblers (du Plessis et al. 2012; Paniw et al. 2019; Van de Ven et al. 2020), and could result from a combination of factors. One possibility is increased energetic expenditure on thermoregulation; many other small mammals have been shown to employ costly mechanisms for homeothermy, such as vasodilation, elevated heart rate, and evaporative cooling (Kamau et al. 1979; Müller and Lojewski 1986; Chalwin‐Milton et al. 2024). Increases in metabolic rate and decreases in water content of prey (both associated with higher temperatures; Gillooly et al. 2001; Tattersall et al. 2012; Everatt et al. 2015), as well as the potential for reduced foraging efficiency (du Plessis et al. 2012; Van de Ven et al. 2020), could further account for reduced body mass gains. Body temperature can also be regulated via suppression of thermogenic activities (e.g., digestion, lactation) in hot conditions (Speakman and Król 2010); banded mongoose pups gain less body mass in hot weather due to such suppression of adult lactation (Khera et al. 2023). Some or all of these processes likely account for our observed reductions in dwarf mongoose body mass gain on hot days, even if foraging and movement levels were maintained. In principle, being away from overnight burrows for longer on hot days, owing to earlier morning departures and later evening arrivals, means that there could be the chance for increased foraging time. However, it is likely that the mongooses simply spent longer inactive in the hottest parts of the day. While we do not have data to test that directly, groups were regularly found at the start of the afternoon session in the same location they were left at the end of the morning session.

Importantly, we demonstrate that despite reductions in body mass gain on hot days, neither adults nor pups show complete compensatory increases in body mass on subsequent cooler days. We are confident that our analyses could detect up to a 2/3 (adults) and 1/3 (pups) regain of the reductions in body mass gain on hot days; however, there was no such compensation on post‐hot days compared to post‐cooler ones. Moreover, body mass consequences are cumulative in adults, with lower evening body masses recorded following three hot days compared to a single one, consistent with previous bird studies (Cunningham et al. 2013; Gardner et al. 2016). In other species, reduced body mass has been linked to shorter dominance tenure (Clutton‐Brock et al. 2006) and reduced survival of both adults and young (Ozgul et al. 2014; Paniw et al. 2019; Bright Ross et al. 2021). As the frequency of hot days is predicted to escalate, reduced body‐mass gains could have increasingly significant life‐history consequences.

The likely direct effects of hot temperatures were not compensated for by many of the potential behavioural changes that we predicted: there were no clear differences between hot and cooler days in the time that dwarf mongoose groups spent inactive, the distance they travelled, and their maximum displacement distance during observed foraging periods. One possibility is that the mongooses do not have the capacity for temperature‐related behavioural plasticity. However, while altering spatiotemporal decision‐making, such as increasing inactivity and reducing movement, is a strategy used to reduce the risk of heat stress in yellow‐billed hornbills ( *Tockus leucomelas* ), American pikas, and African wild dogs (Hall and Chalfoun 2019; Rabaiotti and Woodroffe 2019; Van de Ven et al. 2019), such changes in behaviour can be costly. For instance, greater inactivity comes at the expense of time available for finding food (Hall and Chalfoun 2019; Van de Ven et al. 2019). Reduced movement between patches may induce depletion of resources (Charnov 1976) and exacerbate intragroup competition, reducing group‐living benefits (Randall et al. 2005). Reducing maximum displacement could lessen territory coverage, which may have negative consequences for its defence; in dwarf mongooses, group defence against conspecific rivals relies on cooperative scent‐marking at communal latrines dispersed across the territory (Christensen et al. 2016; Morris‐Drake, Linden, et al. 2021). Such costs could constrain the implementation of spatiotemporal behavioural plasticity predicted in hot weather.

Similarly, while we predicted that a strategy to maximise foraging opportunities at cooler times on generally hotter days would be to reduce the time spent at the burrow before and after daytime foraging excursions, there was no clear difference between hot and cooler days. Dwarf mongooses undertake 90% of all grooming of groupmates in the burrow area (Kern and Radford 2018), with grooming used to strengthen social bonds (Kern and Radford 2021), in cross‐commodity exchanges of cooperative behaviour, such as to reward sentinel contributions (Kern and Radford 2018), and for post‐conflict management (Morris‐Drake, Kern, and Radford 2021). The consistency in the time spent at the burrow on hot and cooler days may suggest that the regulation of cooperation needs to be prioritised. However, if foraging at cooler temperatures (e.g., earlier in the day) is important to offset direct effects of hot temperatures, then foraging should be prioritised over grooming. The lack of such evidence suggests that the mongooses may not be capable of such behavioural plasticity.

The one set of behavioural differences that we found was in burrow‐related timings: dwarf mongoose groups emerged and departed from their overnight burrows earlier in the morning, as well as arriving at and entering their burrows later in the evening on hot days. Previous work has revealed that other species use temporal shifts in activity to reduce overlap with the hottest diurnal periods, likely to minimise the risk of heat stress and the energetic expenditure invested in thermoregulation (Brivio et al. 2024; Funghi et al. 2019; Levy et al. 2019; Rabaiotti and Woodroffe 2019). An alternative explanation for the difference in burrow emergence and entering times of the mongooses relates to the internal temperatures of their refuges. If the burrow reaches a threshold temperature earlier on hotter days, the mongooses may emerge sooner; likewise, they may have to wait until later in the evening for the burrow to cool to an acceptable temperature. Regardless of the exact reason for the difference in timings on hot days, there could be associated costs. For instance, dwarf mongoose groups often arrived at and entered their burrow post‐sunset on hot days, likely increasing their vulnerability to crepuscular predators, such as servals ( *Leptailurus serval* ), black‐backed jackals ( *Canis mesomelas* ) and African spotted eagle owls ( *Bubo africanus* ).

The capacity of a species for behavioural plasticity in response to hot weather is likely indicative of its ability to mitigate the fitness consequences of climate change (Vedder et al. 2013). Whilst dwarf mongooses extend their day with early morning departures and late evening arrivals on hot days, we find little evidence of behavioural plasticity; hence, they were unable to mitigate fully the negative effects on daily body mass gain associated with hot weather. Dwarf mongooses may not have the capacity for temperature‐regulated behavioural plasticity, or constraints, such as trade‐offs with other fundamental behaviours (e.g., grooming and territory defence), may limit the plasticity that can be exhibited. Plausibly, dwarf mongooses may exhibit plasticity outside the scope of our analyses, such as during periods of midday inactivity, which future study could address. Even in conservative projections, the frequency and intensity of hot weather are anticipated to escalate (IPCC 2023; Mbokodo et al. 2020); the mean air temperature in our study area has risen by ca. 2°C in the last 50 years. Emerging evidence also highlights the importance of studying the interactions of temperature and rainfall (Khera et al. 2025; Thorley et al. 2025), which warrants consideration in future work. Furthering our understanding of the responses of wildlife to changing climatic conditions is crucial if we are to understand resilience in the Anthropocene.

## Author Contributions

**Lauren S. Vane:** conceptualization (equal), data curation (equal), formal analysis (equal), investigation (equal), methodology (equal), writing – original draft (equal). **Josh J. Arbon:** data curation (equal), formal analysis (equal), funding acquisition (equal), methodology (equal), project administration (equal), writing – review and editing (equal). **Amy Morris‐Drake:** formal analysis (equal), project administration (equal), writing – review and editing (equal). **Julie M. Kern:** project administration (equal), writing – review and editing (equal). **Andrew N. Radford:** conceptualization (equal), formal analysis (equal), funding acquisition (equal), methodology (equal), project administration (equal), supervision (equal), writing – review and editing (equal).

## Ethics Statement

The work was conducted under permission from the Limpopo Department of Economic Development, Environment and Tourism (permit number: 001‐CPM403‐00013) and ethical approval from the University of Pretoria, South Africa (Animal Ethics Committee: NAS321/2022) and the University of Bristol, UK (Animal Welfare and Ethics Review Body: UIN/17/074).

## Conflicts of Interest

The authors declare no conflicts of interest.

## Supporting information


**Data S1:** ece371963‐sup‐0001‐Supinfo.zip.

## Data Availability

All data and code have been made available within the Supporting Information S1.
